# The discovery of a new lifespan-extending gene in insects

**DOI:** 10.1007/s44297-024-00032-1

**Published:** 2024-07-23

**Authors:** Jacob L. Steenwyk

**Affiliations:** Howards Hughes Medical Institute and the Department of Molecular and Cell Biology, University of California, Berkeley, Berkeley, CA USA

Mitochondria play a critical role in cellular function. For example, cellular adenosine triphosphate (ATP) is generated via the oxidative phosphorylation pathway [1]. The aberrant function of this pathway has been linked to numerous diseases, including age-related ones [2]. The association between oxidative phosphorylation and age has been strengthened by observations that pathway function declines with aging in humans, and defects have been shown to impact longevity [3].

While the mitochondrial matrix is the primary site of ATP synthesis, genes responsible for this process are encoded in both the mitochondria and nucleus (Fig. 1A). In fact, more nuclear-encoded genes contribute to the oxidative phosphorylation pathway than mitochondrial ones (~ 80 to 13, respectively) [4]. Reflecting the shared function of nuclear- and mitochondrial-encoded oxidative phosphorylation genes, these genes tend to coevolve across diverse animal lineages [5].

**Fig. 1:**
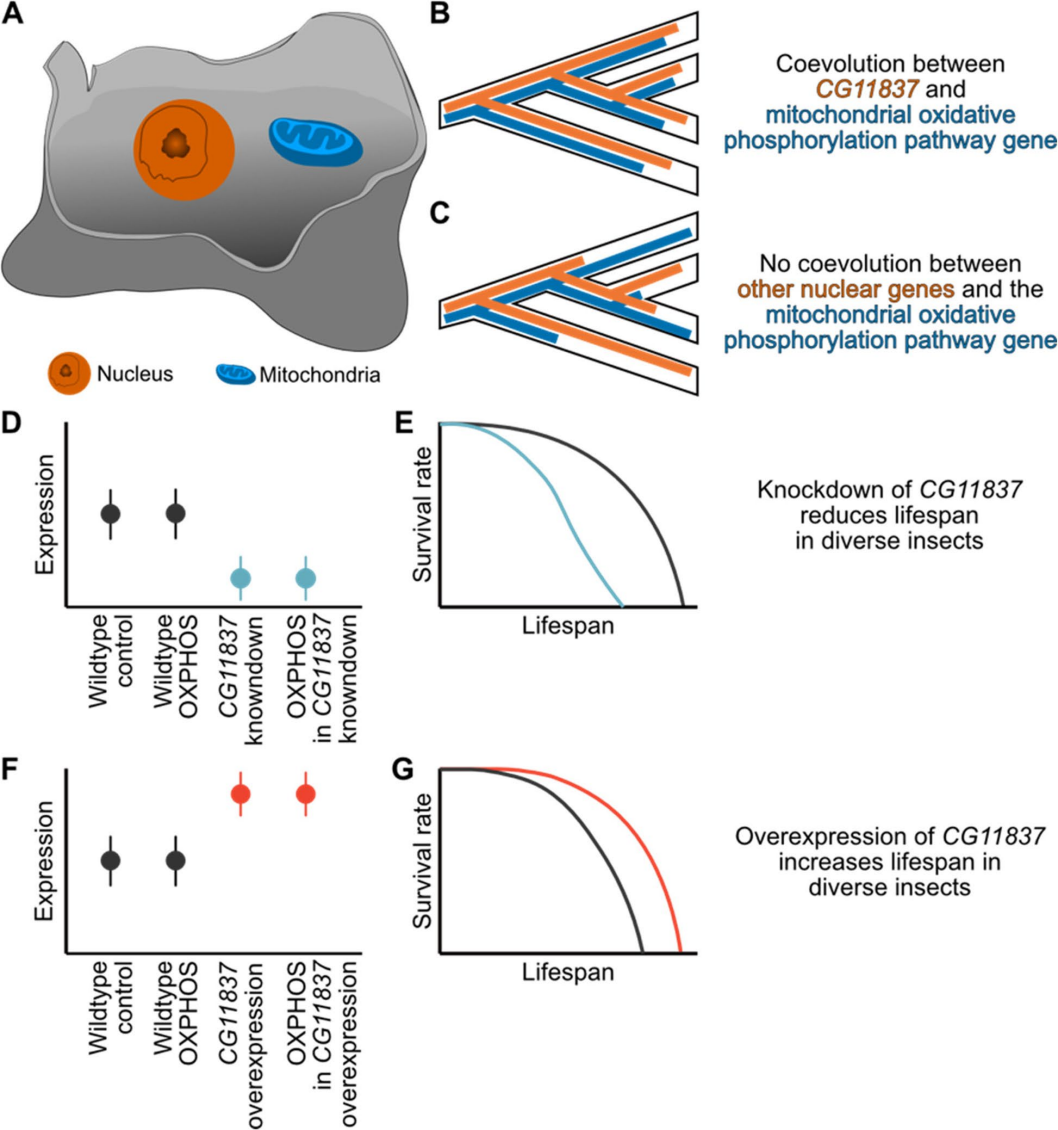
Identification and experimental validation of a new longevity gene. **A** The nucleus and mitochondria are distinct cellular compartments that encode separate genomes. **B** Significant coevolution was observed between nuclear gene *CG11837* and mitochondrially encoded genes comprising the oxidative phosphorylation pathway. **C** In contrast, other nuclear genes did not have signatures of coordinated evolutionary rates. **D** Knockdown of *CG11837* resulted in lower oxidative phosphorylation pathway (OXPHOS) expression. **E** Moreover, reduced expression of *CG11837* resulted in a reduced lifespan in diverse insects. **F** In contrast, overexpression of *CG11837 increased the* expression of OXPHOS and **G** longer-lived insects. A is adapted from an image available to the public domain from Wikimedia Commons

Gene coevolution (or evolutionary rate covariation) is when pairs of genes have coordinated shifts in rates of sequence evolution across speciation events (Fig. 1B) [6]. Significant gene coevolution is often observed among genes that share function, are coexpressed, or are part of the same protein complexes [7]. In contrast, genes that are not coevolving tend to have different rates of sequence evolution (Fig. 1C). Gene coevolution has been used to recapitulate genetic networks, capturing the complexity of gene–gene relationships that confer genomic function and screen for novel gene function [6, 8]. Despite these advances, this approach remains relatively untested to identify novel genes – even non-mitochondrial-targeted genes – that contribute to robust oxidative phosphorylation pathway function.

To address this gap, researchers conducted a comprehensive survey of gene coevolution among nuclear and mitochondrial-encoded genes from 472 insects [9]. This systematic study addresses three questions: (1) What is the landscape of gene coevolution among non-mitochondria-targeted nuclear genes and nuclear-encoded oxidative phosphorylation genes with those encoded in the mitochondria; (2) what non-mitochondria-targeted nuclear genes exhibit robust signatures of gene coevolution with mitochondrial-encoded oxidative phosphorylation genes; and (3) do these genes function in the oxidative phosphorylation pathway?

The analysis identified 75 non-mitochondria-targeted nuclear genes exhibiting strong signatures of gene coevolution with mitochondrial-encoded oxidative phosphorylation genes. Among these, the uncharacterized gene *CG11837* emerged as a gene of interest (Fig. 1B). The association between *CG11837* and the oxidative phosphorylation pathway was also uncovered in transcription analysis. Specifically, *CG11837* knockdown reduced the expression of genes in the oxidative phosphorylation pathway (Fig. 1D). *CG11837* knockdown also resulted in a shortened median lifespan in diverse insect species (Fig. 1E).

In contrast, *CG11837* overexpression increased expression of the oxidative phosphorylation pathway (Fig. 1F) and increased insect lifespan (Fig. 1G). extended the median lifespan of multiple species. In human cells, overexpression of *DIMT1*, the putative *CG11837* human ortholog, helped protect cells from senescence. These findings indicate that *CG11837* is a conserved gene that impacts longevity across multiple species. While the precise mechanism of *CG11837* remains unknown, this study successfully identified a novel, non-mitochondrially targeted gene that contributes to oxidative phosphorylation pathway function and, more broadly, provides a roadmap for genotype-to-phenotype discovery.

These findings have broad potential. For example, *DIMT1* may be a target for certain age-related therapies, as indicated by other researchers [10]; however, more investigation is required. *CG11837* may also be an exciting target for influencing insect pest lifespan. Specifically, reducing *CG11837* expression and possible insect pest lifespan may be possible. While enticing, studies tailored to address this approach's potential strengths and pitfalls are prerequisites.

Together, gene coevolution is an exciting method for gaining insight into potential gene function. Although this article has focused on the recent discovery of a new longevity gene in insects [9], gene coevolution can help uncover genes contributing to nearly any phenotype. Gene coevolution may also be used with other methods to predict shared function, such as coexpression and correlated gene presence/absence patterns. Thus, together with other methods, gene coevolution is an exciting approach that can help address a longstanding goal in biology – what is the function of genes?

## Data Availability

Not applicable.
